# Genome Evolution of Invasive Methicillin-Resistant Staphylococcus aureus in the Americas

**DOI:** 10.1128/spectrum.00201-22

**Published:** 2022-05-31

**Authors:** Joshua T. Smith, Elissa M. Eckhardt, Nicole B. Hansel, Tahmineh Rahmani Eliato, Isabella W. Martin, Cheryl P. Andam

**Affiliations:** a Department of Molecular, Cellular and Biomedical Sciences, University of New Hampshire, Durham, New Hampshire, USA; b Broad Institute of MIT and Harvard, Cambridge, Massachusetts, USA; c Dartmouth-Hitchcock Medical Center and Dartmouth College Geisel School of Medicine, Lebanon, New Hampshire, USA; d Department of Chemical Engineering, University of New Hampshire, Durham, New Hampshire, USA; e Department of Biological Sciences, University at Albany, State University of New York, Albany, New York, USA; University of Pittsburgh School of Medicine

**Keywords:** *Staphylococcus aureus*, bloodstream infection, invasive, genome evolution, methicillin resistance, MRSA, invasive microorganisms

## Abstract

Staphylococcus aureus causes a variety of debilitating and life-threatening diseases, and thus remains a challenging global health threat. S. aureus is remarkably diverse, yet only a minority of methicillin-resistant S. aureus (MRSA) clones have caused pandemic proportions of diseases. The genetic drivers of the successful dissemination of some clones across wide geographical expanses remain poorly understood. We analyzed 386 recently published MRSA genomes from bloodstream infections sampled in North, Central, and South America from 2011 to 2018. Here, we show that MRSA-associated bloodstream infections were attributable to two genetically distinct lineages. One lineage consisted almost exclusively of sequence type (ST) 8, which emerged in 1964. A second lineage emerged in 1986 and consisted of STs 5, 105, and 231. The two lineages have simultaneously disseminated across geographically distant sites. Sublineages rapidly diverged within locations in the early 2000s. Their diversification was associated with independent acquisitions of unique variants of the mobile *mecA*-carrying chromosomal cassette and distinct repertoires of antimicrobial resistance genes. We show that the evolution and spread of invasive multidrug-resistant MRSA in the Americas was driven by transcontinental dissemination, followed by more recent establishment and divergence of local pathogen populations. Our study highlights the need for continued international surveillance of high-risk clones to control the global health threat of multidrug resistance.

**IMPORTANCE** Bloodstream infections due to S. aureus cause significant patient morbidity and mortality worldwide, exacerbated by the emergence and spread of methicillin resistant S. aureus (MRSA). This study provides important insights on the evolution and long-distance geographic expansion of two distinct MRSA lineages that predominate in bloodstream infections in the past 5 decades. The success of these two lineages partly lies on their acquisition of a diverse set of antimicrobial resistance genes and of unique variants of the mobile genetic element SCC*mec* that carries the gene *mecA* conferring resistance to beta-lactams. High-risk antimicrobial resistant clones can therefore rapidly disseminate across long distances and establish within local communities within a short period of time. These results have important implications for global initiatives and local epidemiological efforts to monitor and control invasive MRSA infections and transcontinental spread of multidrug resistance.

## INTRODUCTION

The persistently high morbidity and mortality from diseases caused by Staphylococcus aureus make it a formidable public health threat (1). The bacterium typically colonizes the anterior nares, mucous membrane, and skin, and can be carried asymptomatically. However, it can penetrate into deep tissues and other normally sterile sites in the body when cutaneous and mucosal barriers are disrupted (e.g., due to wounds, invasive medical devices, or chronic skin conditions) (2). S. aureus causes a wide range of infections, ranging from superficial skin infections to invasive diseases such as pneumonia, septicemia, infective endocarditis, osteomyelitis, and various toxicoses such as toxic shock syndrome (2). S. aureus is the second most common cause of bloodstream infection and is the most important cause of associated deaths (3, 4). Estimates of disease incidence of S. aureus-associated bloodstream infections range between 10 to 30 cases per 100,000 person-years, while hospital mortality ranges between 15% and 40% (5–7). In the United States alone, nearly 120,000 S. aureus bloodstream infections and 20,000 associated deaths occurred in 2017 (8). Of critical concern is the increasing proportion of bloodstream infections caused by methicillin-resistant S. aureus (MRSA). MRSA complicates the treatment of bloodstream infections because prompt source control and prolonged antimicrobial therapy are critical (9). Even more worrisome is that MRSA strains are resistant not only to nearly all beta-lactams, but many strains are often also resistant to multiple other antimicrobial classes (2).

A minority of emergent MRSA clones have disseminated globally and have caused pandemic proportions of infections in both health care and community settings (10). The epidemiology of MRSA infections has been characterized by sequential and sometimes overlapping epidemic waves of replacement of one predominant clone by another (11). Six major waves have been recognized, with the first wave occurring in the mid-1940s as penicillin-resistant strains increased in hospitals where the selective pressures for resistance are greatest (11). The specific clones that drive each epidemic wave vary with geographical location, origins (hospital, community, or livestock), and rates and types of infections (11, 12). However, the genetic drivers of the successful dissemination of some high-risk MRSA clones across wide geographical expanses remains poorly understood.

Here, we aim to elucidate the evolutionary history and long-range geographic spread of invasive MRSA. We leveraged previously published genomic data sets of MRSA isolates from bloodstream infections (*n* = 386 genomes) sampled from North, Central, and South America spanning a sampling period of 8 years. Our data revealed that S. aureus-associated bloodstream infections were attributable to two genetically distinct lineages that emerged independently in 1964 and 1986. The diversification of each of the two dominant lineages was associated with independent acquisitions of unique variants of the mobile *mecA*-carrying chromosomal cassette and distinct repertoires of mobile antimicrobial resistance genes. Overall, the evolution and spread of invasive multidrug-resistant MRSA in the Americas was driven by transcontinental dissemination, followed by more recent establishment and divergence of local pathogen populations.

## RESULTS

### Two distinct MRSA clonal lineages dominate the S. aureus population in bloodstream infections.

We compiled a total of 386 previously published *de novo* assembled genomes from either Illumina shotgun or PacBio SMRT sequencing data of MRSA isolates obtained from bloodstream infections (Fig. S1; Table S1). These came from three states in the United States: Minnesota (*n* = 48 genomes) (13), New Hampshire (*n* = 102) (14), and New York (*n* = 131) (15), as well as nine countries from Central and South America (*n* = 105) (16). The latter group included Argentina (*n* = 8), Brazil (*n* = 13), Chile (*n* = 16), Colombia (*n* = 16), Ecuador (*n* = 12), Guatemala (*n* = 18), Mexico (*n* = 5), Peru (*n* = 8), and Venezuela (*n* = 9). Combined, the genomes represented samples collected between 2011 and 2018.

**FIG 1:**
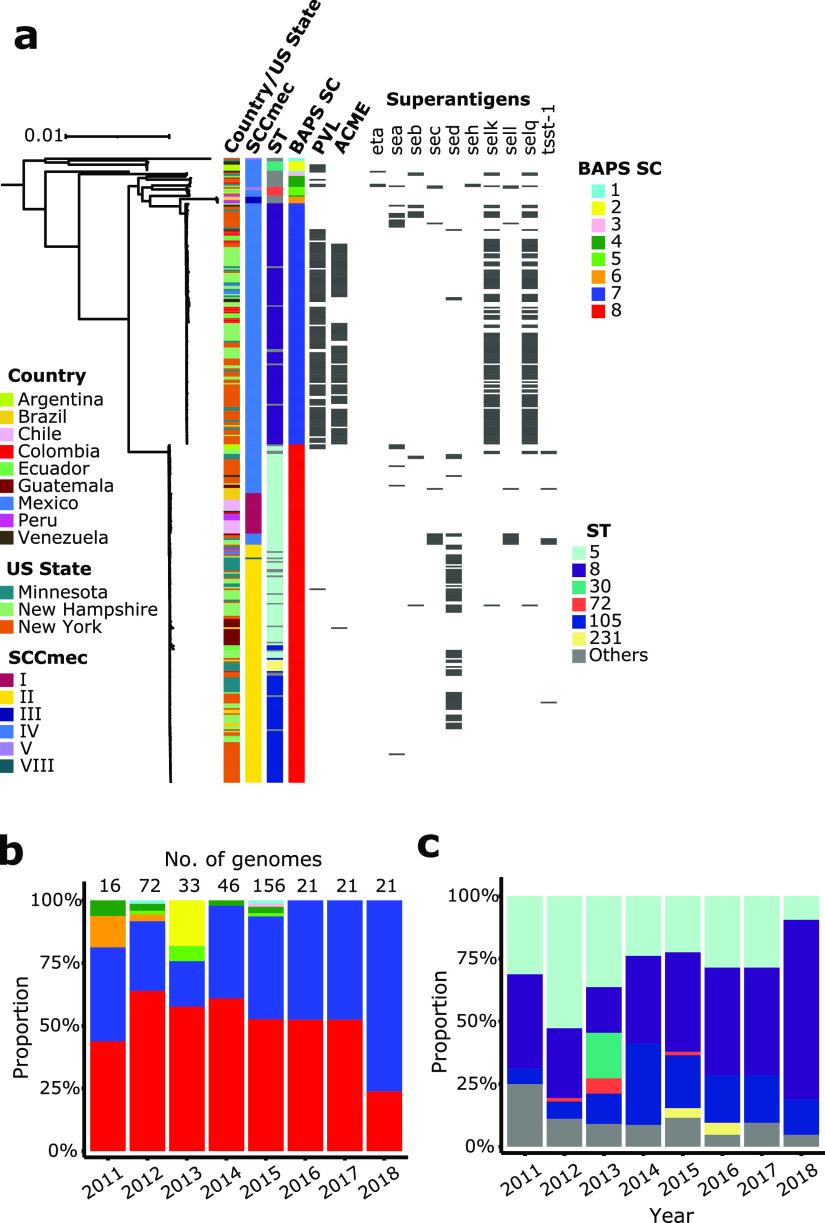
Phylogenetic relationship and annual distribution. (a) Midpoint-rooted maximum likelihood tree showing phylogenetic structure of 386 MRSA isolates. Scale bar represents the number of nucleotide substitutions per site. Matrix shows the presence/absence of superantigen genes, with gray blocks representing the presence of the gene. BAPS SC refers to sequence clusters identified by RhierBAPS. Yearly distribution of sequence clusters (b) and sequence types (ST) (c) throughout the study period.

Assembled genomes were annotated, revealing a total of 4,907 clusters of orthologous genes (COGs). Of these, a total of 2,173 COGs were present in 99% to 100% of the genomes, making up the core genome of the data set (Fig. S2; Table S2). Population structure analysis using Bayesian hierarchical clustering of the core genome alignment showed eight distinct sequence clusters, ranging in size from two to 209 genomes (Fig. 1a). Phylogenomic analysis revealed the presence of two dominant lineages. These were labeled as sequence clusters 7 and 8, consisting of 149 (38.6% of the total population) and 209 (54.1% of the total population) genomes, respectively (Fig. 1a, b). We carried out *in silico* multilocus sequence typing, a method which partitions the population based on allelic variation in seven housekeeping genes and assigns a sequence type (ST) to strains (17) (Fig. 1c). These STs can be more broadly categorized into clonal complexes (CC) when at least five of seven alleles are shared. Sequence cluster 7 consisted entirely of CC8 and almost exclusively of ST8 (*n* = 143 genomes, making up 96.0% of sequence cluster 7), while sequence cluster 8 consisted entirely of CC5 and mostly of STs 5 (*n* = 115 genomes), 105 (*n* = 69 genomes), and 231 (*n* = 7 genomes), making up 55.0%, 33.0%, and 3.3% of sequence cluster 8, respectively. In the context of well-known pandemic strains, sequence cluster 7 is most closely related to MRSA strain USA300, which arose in the early 1990s in the United States and commonly spreads in the community (18–20). This lineage frequently harbors the genetic components arginine catabolic mobile element (ACME), which plays a role in bacterial growth, colonization and survival inside the host, and Panton-Valentine leukocidin (PVL), which causes leukocyte lysis and tissue necrosis (10, 19, 21). Across our data set, 119/131 genomes carrying PVL and 98/99 genomes carrying ACME were found in sequence cluster 7 (Fig. 1a). Sequence cluster 8 is most closely related to MRSA strain USA100, a highly invasive strain primarily associated with hospital infections (20, 22). In contrast with sequence cluster 7, only four PVL-positive genomes and one ACME-positive genome were found in sequence cluster 8 (Fig. 1a). The remaining six sequence clusters were each composed of two to eight genomes and comprised low frequency genotypes that were highly divergent from the rest of the population and from each other.

We next sought to investigate the phylogenetic distribution of genes that encode superantigens. Superantigens comprise a family of potent immunostimulatory exotoxins that modulate the immune system by stimulating dysregulated T-cell proliferation (23, 24) and are known to contribute to a variety of staphylococcal diseases (23, 24), including bloodstream infections (25). Of the 29 known superantigen types (23, 24), we detected a total of 10 superantigens in the American MRSA population that were differentially distributed between the two dominant lineages (Fig. 1a, Fig. S3; Table S3). The most prevalent were *sed*, *selK*, and *selQ.* The plasmid-borne *sed* gene was found in 73 genomes, of which 70 are from sequence cluster 8. In contrast, the staphylococcal enterotoxin-like (*sel;* lacking emetic properties) genes *selK* and *selQ* were detected mostly in sequence cluster 7 (109 genomes out of a total of 115 *selK*- and *selQ*-positive genomes). Other less common superantigen genes include *eta*, *sea*, *seb*, *sec*, *seh*, *selI*, and *tsst-1*. The *tsst-1* gene is carried by a pathogenicity island and encodes the toxic shock syndrome toxin 1. We detected *tsst-1* in three different STs within sequence cluster 8 (ST 5, ST 105, and ST 840).

### Different types of the mobile *mecA*-carrying chromosomal cassettes distinguishes the two MRSA lineages.

The mobile element staphylococcal cassette chromosome (SCC*mec*) carries the *mec* complex consisting of the *mecA* gene, which confers resistance to almost all beta-lactam antimicrobial agents as well as the regulatory elements *mecI* and *mecR* (26, 27). There are currently 14 structural variants of SCC*mec* based on the combination of the *mec* gene complex and the recombinases (*ccr*) (26). In the American MRSA population, the two dominant lineages carry distinct sets of SCC*mec* types (Fig. 1a; Table S1). Sequence cluster 7 was entirely made up of SCC*mec* type IV. Type IV is 21Kb in size, the smallest among the SCC*mec* types, and does not contain other resistance genes except *mecA*. In contrast, sequence cluster 8 consisted of SCC*mec* types I (25 genomes), II (146 genomes), IV (37 genomes), and VIII (one genome). The 34Kb type I contains 22-bp invert repeat sequences and 41 coding DNA sequences, of which 36 are hypothetical open reading frames (26, 27). Type II is 53Kb in size and contains four repeat regions, three mobile elements, 51 coding DNA sequences (with 33 that are hypothetical), an integrated copy of plasmid pUB110 that encodes resistance genes *ant* (kanamycin and tobramycin) and *ble* (bleomycin), gene encoding the recombination enzyme for pUB110, and the transposon Tn554 carrying resistance genes *ermA* (erythromycin) and *spc* (spectinomycin) (26, 27). Type VIII is approximately 32Kb in size and contains the transposon Tn554, which harbors the genes *ermA* (conferring resistance to streptogramin, macrolide, and lincosamide), *aad9* (aminoglycoside resistance), and three genes encoding transposases (26, 27).

### Genetic basis of multidrug resistance varies between the two lineages.

**FIG 2:**
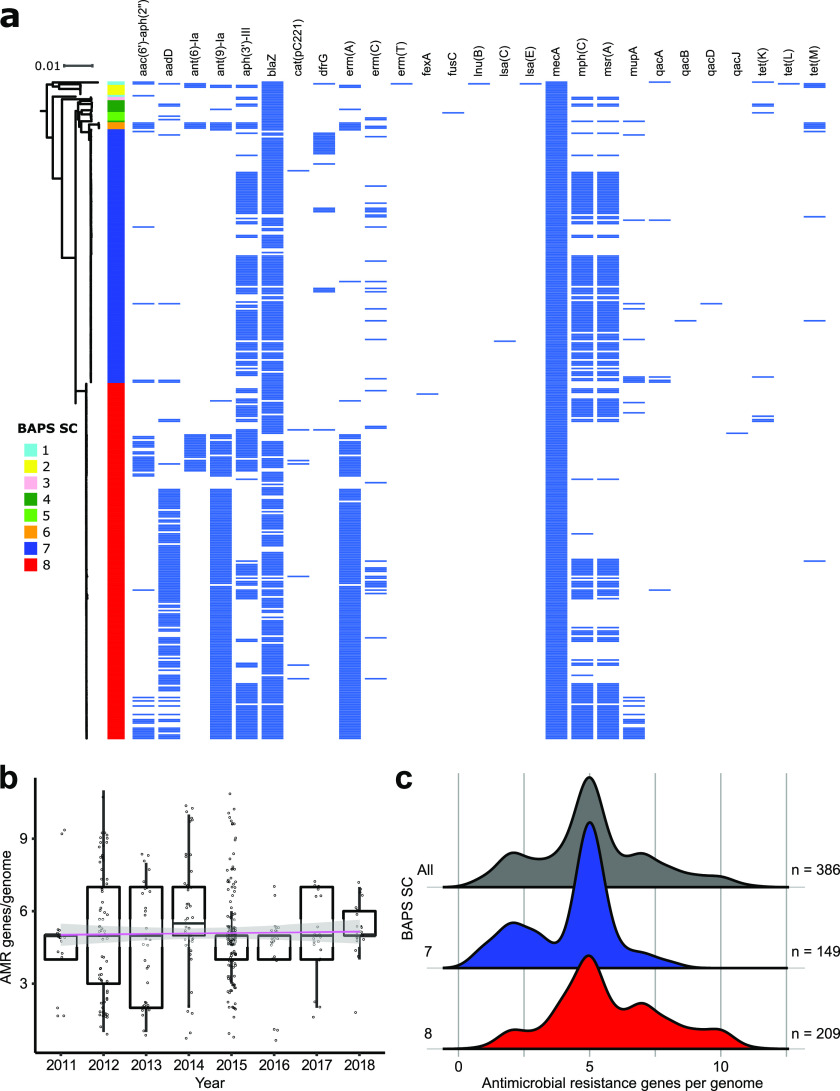
Distribution of antimicrobial resistance genes. (a) Phylogenetic distribution of acquired antimicrobial resistance genes identified using ABRricate and ResFinder. (b) Number of antimicrobial resistance genes per genome per year with predicted linear regression over time plotted in pink. Median values for each year marked in thicker horizontal black lines. (c) Density plots comparing antimicrobial resistance genes per genome for the entire data set and for each of sequence clusters 7 and 8.

Other non-*mecA* antimicrobial resistance genes may also be present in MRSA, which may result in the emergence of multidrug resistance. We therefore considered bioinformatic evidence to examine the presence of horizontally acquired antimicrobial resistance genes in our data set (Fig. 2a; Table S4). Despite the differences in the number of genomes collected per year, we found that the number of acquired antimicrobial resistance genes per genome remained consistent over 8 years (Fig. 2b). Across the entire MRSA population, individual genomes carried five resistance genes on average. The number of resistance genes per genome in sequence cluster 7 was five (range = 1 to 8; Fig. 2c), with the most commonly detected non-*mecA* resistance genes aph(3′)-III (*n* = 91 genomes, corresponding to 61.1% of the genomes in the sequence cluster), *blaZ* (*n* = 132; 88.6%), *mphC* (*n* = 97; 65.1%), and *msrA* (*n* = 97; 65.1%). In sequence cluster 8, we also detected five resistance genes per genome (range = 1 to 11; Fig. 2c). The most common non-*mecA* resistance genes in sequence cluster 8 were *aadD* (*n* = 115 genomes, corresponding to 55.0% of the genomes in the sequence cluster 8), ant(9)-Ia (*n* = 170; 81.3%), *blaZ* (*n* = 177; 84.7%), *ermA* (*n* = 169; 80.9%), *mphC* (*n* = 82; 39.2%), and *msrA* (*n* = 79; 37.8%).

Two genomes were particularly notable because they harbor resistance genes not found in other strains. Genome SRR5486295 (ST398) from Minnesota carried 11 resistance genes and is the only genome carrying *ermT* (streptogramin, macrolide and lincosamide resistance), *lnuB* (lincosamide resistance), *lsaE* (multidrug resistance [lincosamide, phenicol, pleuromutilin, tetracycline, oxalidinone, streptogramin, macrolide]), and *tetL* (tetracycline resistance). The gene *fusC* (fusidic acid resistance) was found only in genome GCA_008936655.1. This ST72 isolate was obtained from New York in 2015 and is one of two genomes carrying SCC*mec* Type Vc. While less common, the existence of these resistance genes should still be a cause for concern because they can still be mobilized via horizontal gene transfer (28) and may potentially increase in frequency in the population.

### Temporal origins and population dynamics of the two MRSA lineages.

**FIG 3:**
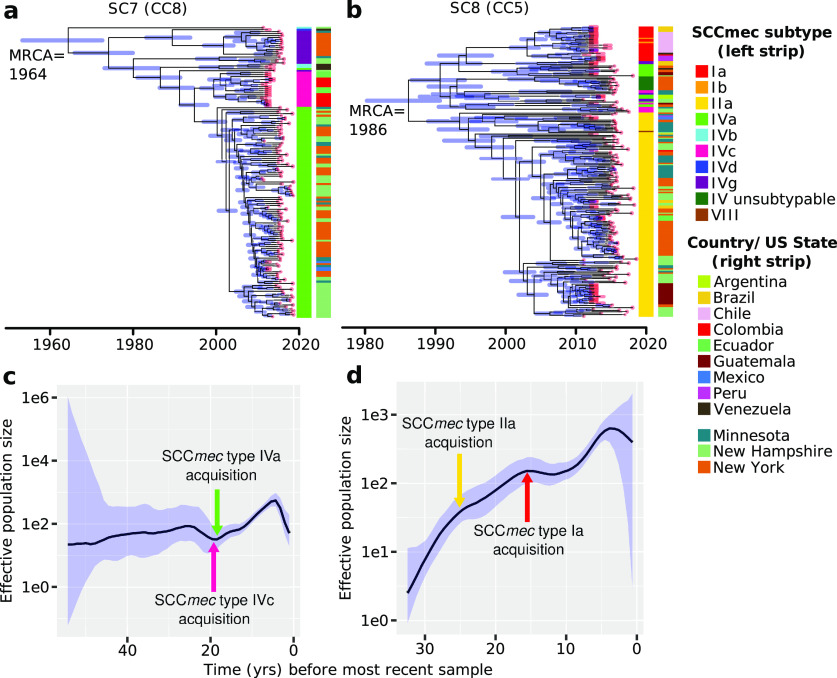
Bayesian phylogeny and population dynamics of sequence clusters 7 and 8. (a, b) Bayesian maximum clade credibility time-calibrated phylogenies based on nonrecombining regions of the core genome. Divergence date (median estimate with 95% highest posterior) is indicated by internal tree nodes. Blue horizontal bars at each node represent 95% confidence intervals. Red horizontal bars on branch tips represent the time frame in which the sample was obtained (year, month, or date). (c, d) Bayesian skygrowth plots showing changes in effective population size (Ne) over time. Median is represented by a black line and 95% confidence intervals are in blue. MRCA, most recent common ancestor.

To provide a historical perspective on the emergence of the two dominant MRSA bacteremia lineages in the Americas, we constructed time-calibrated phylogenies of sequence clusters 7 and 8. Using BactDating (29), we observed a small but significant positive correlation between the dates of isolation and root-to-tip distances for both sequence clusters (R^2^ = 0.28 and *P* < 1e-4 in sequence cluster 7; R^2^ = 0.20 and *P* < 1e-4 in sequence cluster 8) (Fig. S4), indicating the presence of a clock-like signal. Given the presence of a temporal structure, we performed a dated coalescent phylogenetic analysis. Results show that the two lineages emerge in the Americas at different times (Fig. 3a, b). We estimated the time to the most recent common ancestor (tMRCA) of sequence cluster 7 (CC8) to be 1964 (95% highest posterior density [HPD] intervals:1953 to 1973). The tMRCA of sequence cluster 8 (CC5) was 1986 (95% HPD intervals:1980 to 1991). However, because of disparities in the number of genomes from each location, it may not be possible to precisely define the geographical origins of each sequence cluster.

For each MRSA sequence cluster, we also estimated the change in the effective population size (Ne) (Fig. 3c, d), which is a measure of the rate of change in population composition due to genetic drift (30). The Ne of sequence cluster 7 remained steady after it first emerged. After a slight dip, its Ne rapidly rose ~20 years ago. This increase coincided with the acquisition of two different subtypes of SCC*mec* IV. SCC*mec* subtypes are defined by the variations in other non-*ccr* and non-*mec* parts of the cassette (26, 27). Within sequence cluster 7, we estimate that the sublineage containing SCC*mec* subtype IVc emerged in the South American countries Colombia and Ecuador in 1999 and remained restricted to that region. In 2000, a second sublineage carrying SCC*mec* subtype IVa emerged and spread in the United States and Mexico. Finally, we detected another sublineage carrying SCC*mec* subtype IVg, that emerged in New York in the early to mid-2000s. Subtype IVc carries the transposon Tn4001, while IVa carries an integrated plasmid pUB110 (26, 27). Subtype IVg carries three staphylococcal enterotoxin genes (31).

The Ne of sequence cluster 8 rapidly increased after its emergence. During this time, it acquired different SCC*mec* subtypes. The sublineage carrying SCC*mec* subtype IIa is geographically widespread and is found across North, Central, and South America. Notable is a small clade that rapidly diversified in Guatemala in 2009. Another sublineage that acquired SCC*mec* subtype I emerged in 2002 and is found only in South America. Both subtypes Ia and IIa carry the plasmid pUB110 (26, 27). Finally, a sublineage carrying an SCC*mec* type IV with unknown subtype appeared in 2004 in New York. For both lineages, there is evidence of a decline in the Ne of both lineages in 2013 onwards.

**FIG 4:**
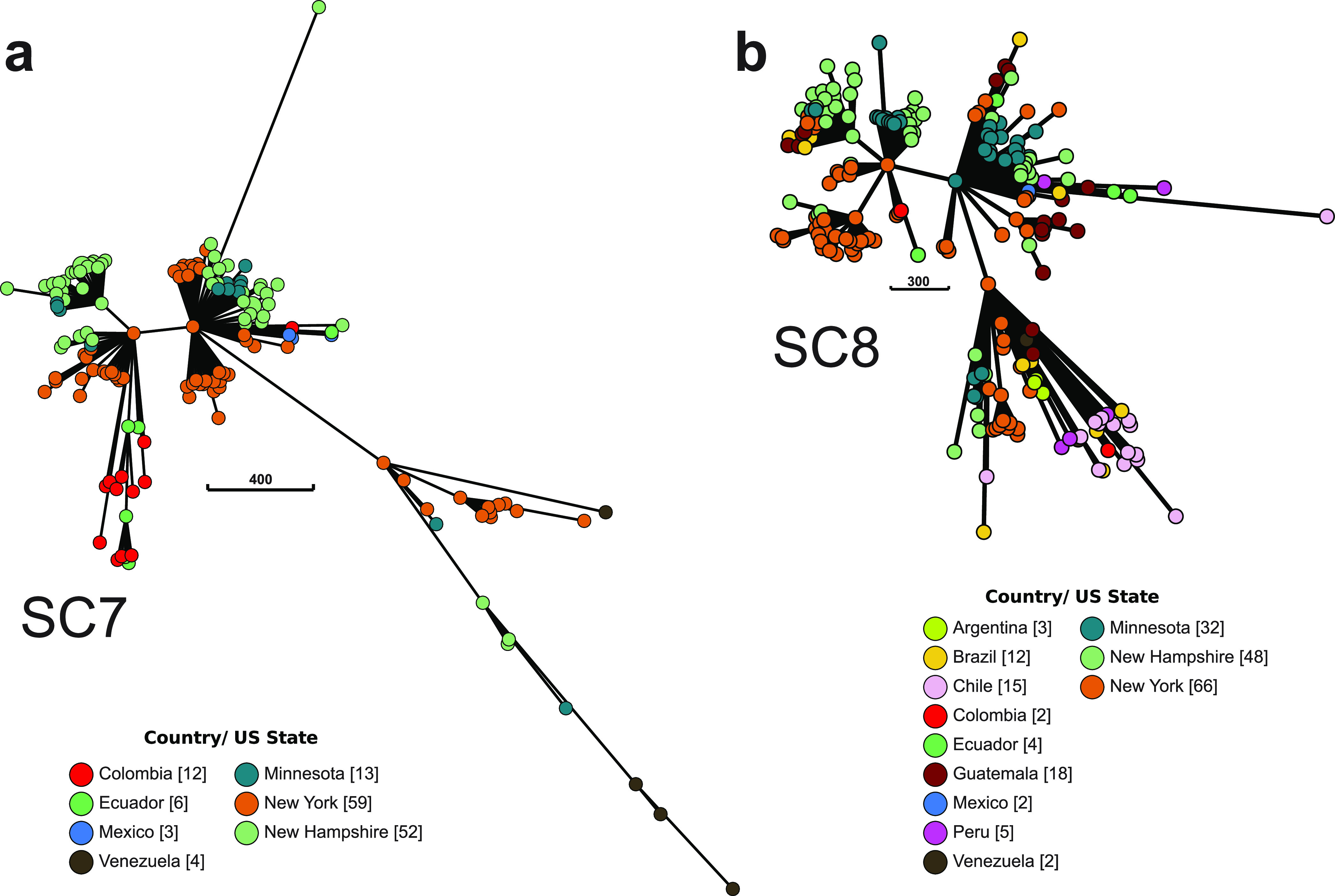
Minimal spanning trees of sequence clusters 7 (a) and 8 (b) based on allelic variation in the core genome alignments. Each node represents isolates with distinct allelic profiles. Node colors represent the geographical origins of the strains. The number in brackets next to location name in the color legend indicates the total number of isolates from that country. Edge scale refers to the number of SNPs and the length of the edge is proportional to the number of SNP differences.

Lastly, we examined the genetic relationships of invasive MRSA using minimum spanning trees, which calculates arithmetic distance matrices based on the degree of dissimilarity among individuals (32, 33). In molecular epidemiology, this path is interpreted as the most feasible chain of pathogen transmission and spread (32, 33). Using the core genome alignments of each of the two major sequence clusters, we show that there was simultaneous expansion of sublineages in multiple locations (Fig. 4). There was relatively little structure related to sampling location, but this is most likely due to the highly disproportionate number of samples from different sites. Both sequence clusters 7 and 8 exhibited a radial branch structure and were each derived from two ancestral nodes. Lastly, most of the stepwise accumulations of SNPs from the ancestral nodes were represented by one of the extant isolates, indicating long persistence of genetic variants through time.

## DISCUSSION

In the 80 years since it was first recognized, MRSA has proven its remarkable ability to rapidly evolve following encounter with antimicrobials and to quickly disseminate across the globe (11). MRSA causes a variety of debilitating and life-threatening diseases, and thus remains a challenging global health threat (1, 2). Using 386 previously published MRSA genomes derived from bloodstream infections, we elucidated their evolutionary history and geographical spread across the American continents. There are two major findings in this study. First, MRSA-associated bloodstream infections were attributable to two genetically distinct lineages that emerged independently, disseminated across continents, and have diversified locally. Second, the success of the two dominant MRSA lineages lies in part on the distinct repertoires of antimicrobial resistance genes and distinct types and subtypes of the mobile SCC*mec* they carry.

Our findings greatly expand the observations from previous studies of STs 5 and 8 sampled from 1980s to 2013 which showed the progressive acquisition of virulence and resistance genes during their expansion in the Americas (19, 34–37). These previous studies have estimated tMRCAs for ST5 and ST8 in the Western Hemisphere to be 1938 and 1940, respectively. Following these periods, sublineages arose in North and South America over the second half of the 20th century. While previous investigations included S. aureus isolates that were also methicillin-susceptible, obtained from different infection sources, and globally distributed, our MRSA bacteremia-focused analyses of tMRCA estimates within the late 20th century are strongly supported. Our analyses also included more recent samples that revealed new insights into their population dynamics. While both sequence clusters have been simultaneously circulating and expanding in the past 5 decades, we observed a decline in their effective population size beginning in 2013 that needs to be closely monitored. In the United States, rates of hospital-onset MRSA bloodstream infections decreased between 2005 and 2012, although the decline slowed between 2013 and 2016 (8). It has been posited that improvements in infection-control efforts in hospitals might have contributed to this decline (8), and this may explain the observed decline at least in the United States. It is also possible that other low-frequency clones have started to increase in frequency during this time, competing with the dominant lineages albeit still undetected. Local clonal expansion followed by replacement of contemporary lineages was previously reported in S. aureus ST239 by STs 5 and 228 in Hungary (38) and by ST59 in China (39). We did not find evidence of replacement by rare genotypes from our data set; however, this may be due to the sampling strategies used in our data set that that could have introduced biases in characterization of common and rare lineages.

Our findings indicate that the diversification and mobility of accessory genes of international clones partly reflects region-specific patterns of selective pressures (e.g., antimicrobial usage), which may influence their fitness and further spread after introduction to different locations. The mobile SCC*mec* can be transferred remarkably fast, even between MRSA and methicillin-susceptible strains during a hospital outbreak (40). In our study, the successive dissemination and replacement of SCC*mec* subtypes in both lineages in the past 5 decades is particularly intriguing. Smaller SCC*mec* types tend to have a fitness advantage (41), and thus the spread of the six subtypes of SCC*mec* IV may be a contributing factor to the success of both sequence clusters. Their rapid mobility is also likely driven by region-specific selective pressure of clinical drug use. Other mobile genetic elements of S. aureus have also been reported to exhibit strong geographic structure, as in the case of the temperate bacteriophage carrying the exfoliative toxin A (42). Moreover, the geographical spread of the American MRSA may lie partly on the coselection and mobility (43) of resistance genes, which requires careful long-term monitoring.

We acknowledge the limitations of the study, which stems from the risk of bias in our sampling strategy due to use of existing data sets. The inadequate number of genome sequences of bloodstream-associated MRSA from Central and South America is a major limitation. The paucity of data from several countries in this region means it is possible that unique or less prevalent genotypes are circulating yet remain invisible to current phylogenomic analyses. Moreover, uneven sampling between geographical regions (either between countries or between individual states in the United States) did not allow us to infer the direction of transmission between locations. The small but significant positive correlation between the dates of isolation and root-to-tip distances is partly due to missing samples and/or under-sampling, especially of older or more historical isolates. A systematic and collaborative cross-country effort to sequencing, surveillance, and monitoring is therefore critical to accomplishing a more rigorous coalescence analysis and precise inference of tMRCA of major bacteremia-causing S. aureus lineages. Notwithstanding these limitations, we obtained sufficient representation of bloodstream-associated MRSA in different parts of the Americas that can be used as a basis for future genomic epidemiology studies of S. aureus bacteremia. This would address lingering questions on patterns of inter- and intracountry transmission networks and hot spots, as well as identification of epidemiologic and health care drivers to S. aureus bacteremia. Lastly, we only focused only on bloodstream infections and therefore the global population structure of MRSA causing other invasive infections might show unique patterns of geographical distribution and evolutionary trajectory.

We conclude that the MRSA population causing bloodstream infections in the Americas was shaped mainly by the clonal expansion, contemporaneous circulation, and rapid geographic spread of two distinct lineages. These results have important implications for the development of effective and robust strategies to effective management of invasive MRSA infections and transcontinental spread of multidrug resistance. Global initiatives and sustained local efforts to monitor the emergence of high-risk clones across geopolitical boundaries are urgently needed.

## MATERIALS AND METHODS

### Data set.

We retrieved a total of 386 previously published genome sequences and associated metadata of MRSA isolates obtained from bloodstream infections from different parts of the American continents. This data set was curated to include assemblies reaching sufficient sequence quality metrics (as described in the next section). We also only included data sets with time (month, year) of isolation from patients. Genome sequences were derived from isolates sampled in Minnesota, USA (*n* = 48 genomes, BioProject: PRJNA384623) (13), New Hampshire, USA (*n* = 102, BioProject: PRJNA673382) (14), New York, USA (*n* = 131, BioProject: PRJNA470993) (15), and nine countries from Central and South America (*n* = 105, BioProject: PRJNA291213) (16). The latter group included Argentina, Brazil, Chile, Colombia, Ecuador, Guatemala, Mexico, Peru, and Venezuela. In all, the genomes represented samples obtained between 2011 and 2018. While exact methicillin resistance testing varied slightly between studies, at a minimum, all isolates were confirmed as resistant using either agar dilution or automated broth microdilution with oxacillin. Results were interpreted according to the Clinical and Laboratory Standards Institute (CLSI) guidelines (44). The sequences were downloaded from the National Center for Biotechnology Information (NCBI) Reference Sequence and Sequence Read Archive databases.

### Genome assembly, annotation, and pan-genome construction.

We used the assembly pipeline program Shovill v.1.1.0 (https://github.com/tseemann/shovill) with the –trim option to yield high-quality genomes. Shovill implements several steps to improve assemblies, including read subsampling to a reasonable depth of 150x, read error correction, trimming adaptor sequences, detecting and removing sequencing errors, and assembling using SPAdes (45). The assemblies were then improved further with scaffolding and gap-closing using SSPACE (46) and GapFiller (47). Genome quality was assessed for all assemblies using QUAST (48) and CheckM (49) with cutoff thresholds of >200 contigs, <40,000 base N50, <90% completeness, and >5% contamination as exclusion criteria. Genomes were annotated using Prokka v.1.14.6 (50). The pan-genome of the data set was assessed using Panaroo v.1.2.7, which uses a graph-based approach using gene neighborhoods combined with gene clustering (51). Panaroo also detects annotation errors and filters sequence contamination (51). We used the option –strict to ensure only high-quality genes were included. Nucleotide sequences were aligned using the program MAFFT (52).

### Phylogenetic and clustering analysis.

Genes present in ≥ 99% of genomes (i.e., core genes) were concatenated to produce a core genome alignment, which was used to build a maximum likelihood phylogenetic tree with IQTree v.2.0.3 (53). We used the ModelFinder algorithm to search the best-fitting nucleotide substitution model (54). Based on the results of ModelFinder, we used a general time reversible model (55) of nucleotide substitution and a FreeRate model of rate heterogeneity with three categories. We also used the Ultrabootstrap software UFBoot2 with 1,000 bootstrap replicates (56) implemented in IQTree. Phylogenetic trees were visualized using the online platform Interactive Tree of Life (IToL) (57). We carried out genetic population structure analysis by partitioning the strains into sequence clusters consisting of genetically similar individuals using the Bayesian hierarchical clustering algorithm RhierBAPS v.1.1.2 (58) with the core genome alignment as input. Single nucleotide polymorphisms (SNPs) from the core genes were identified and aligned using Snippy v.4.6.0 (https://github.com/tseemann/snippy). We used the SNPs from the core genome alignment of each sequence cluster as input in building minimum spanning trees using Grapetree (59). A minimum spanning tree represents a set of edges that link nodes by the shortest possible genetic distance (59), and are thus useful when examining relationships of closely related strains that have undergone short-term divergence (32).

### *In silico* identification of sequence type, SCC*mec*, antimicrobial resistance, and virulence genes.

Sequence type (ST) of each strain was determined using mlst v.2.19.0 (https://github.com/tseemann/mlst), a program which extracts seven single-copy housekeeping genes (*arcC*, *aroE*, *glpF*, *gmk*, *pta*, *tpi*, *yqiL*) and compares their sequence identity to previously deposited allele combinations in the S. aureus PubMLST database (https://pubmlst.org/organisms/staphylococcus-aureus/). To detect horizontally acquired antimicrobial resistance and virulence genes, we used ABRicate v.1.0.0 (https://github.com/tseemann/abricate) using threshold values of >95% sequence identity and >95% sequence coverage to known resistance and virulence genes deposited in the ResFinder database (60) and Virulence Factor Database (VFDB) (61), respectively. We also included all seven distinct ACME types obtained from the VirulenceFinder database (62) and added to the VFDB sequences. With these additions, VFDB contained reference sequences for all superantigens, PVL, and ACME. Lastly, we used staphopia-sccmec v.1.0.0 (https://github.com/staphopia/staphopia-sccmec) to characterize the types and subtypes of SCC*mec* (63).

### Time-calibrated phylogeny and population demographic analyses.

SNP alignments of each major sequence cluster were mapped to reference genomes USA500 (NCBI accession no. GCF_000746505.1) and JH1 (NCBI accession no. GCF_000017125.1). S. aureus strain USA500 is a highly virulent strain of CC8 obtained from a human wound but with systemic infection capability in murine models (64). S. aureus strain JH1 is a strain of CC5 obtained from the bloodstream of a patient with congenital heart disease (65). Using the recombination-free phylogenies generated by GUBBINS (66) for each sequence cluster, we used BactDating v.1.1, a Bayesian framework for estimating the molecular clock rate and coalescent rate (29). We carried out a root-to-tip linear regression analysis and calculation of the coefficient of determination (R^2^) to assess the significance of the temporal signal based on random permutations of sampling dates. When a significant positive correlation between the dates of isolation and root-to-tip divergence was observed, we ran BactDating to infer the dates when common ancestors are estimated to have existed (29). We used a mixed clock model and 10^8^ iterations to conduct molecular dating of the nodes of the tree. We removed the first half of iterations as burn-in and subsequently sampled every 100 iterations. We used Skygrowth v.0.3.1 to estimate the changes in effective population size over time (67).

Default parameters were used for all programs unless otherwise noted.

### Data availability.

NCBI accession numbers, associated metadata, and reference for each genome included in this study are listed in Table S1. All other data supporting the findings of this study are available in the supplementary information files or from the corresponding author upon request.
