# Supplementary material for: Genome Evolution of Invasive Methicillin-Resistant Staphylococcus aureus in the Americas
Source: Microbiol Spectr. 2022 May 31;10(3):e00201-22. doi: 10.1128/spectrum.00201-22 (PMC9241880; doi:10.1128/spectrum.00201-22)
Supplement: Supplemental file 1 — Supplemental material. Download spectrum.00201-22-s0001.pdf, PDF file, 1.4 MB [file spectrum.00201-22-s0001.pdf]

Supplementary information to:

Genome evolution of invasive methicillin-resistant *Staphylococcus aureus* in the Americas

Joshua T. Smith, Elissa M. Eckhardt, Nicole B. Hansel, Tahmineh Rahmani Eliato, Isabella W. Martin, Cheryl P. Andam

Supplementary tables:

Supplementary Table S1. Genome assembly, MLST, BAPS clusters, genetic characteristics, accession numbers and associated metadata of 386 MRSA isolates from bacteremia

Supplementary Table S2. Distribution of genes in the MRSA pan-genome identified using Panaroo

Supplementary Table S3. Distribution of virulence genes identified using ABRricate and VFDB

Supplementary Table S4. Distribution of acquired antimicrobial resistance genes identified using ABRricate and ResFinder

Supplementary figures:

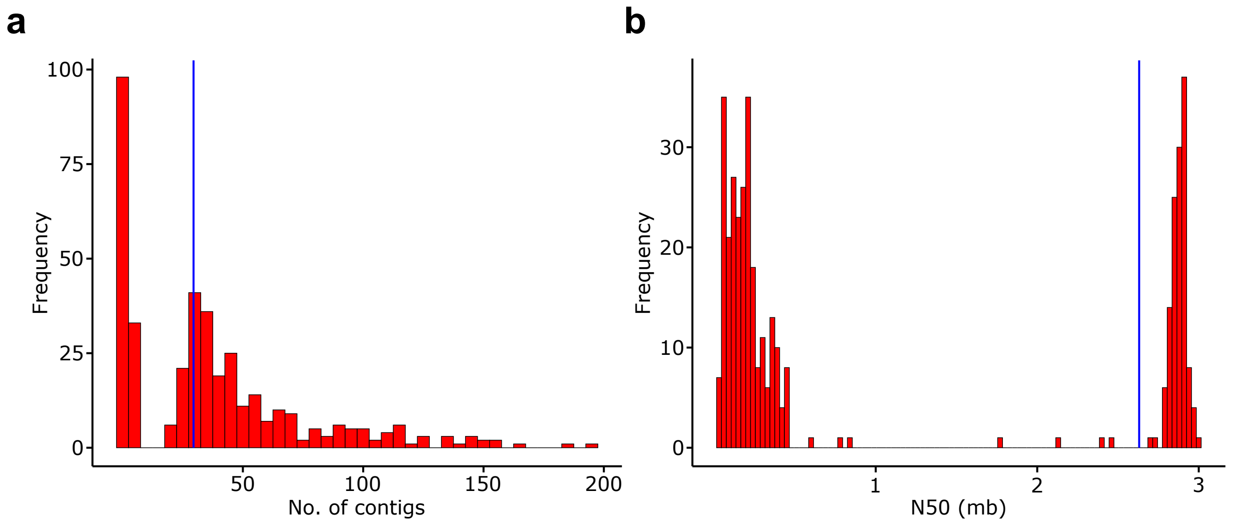

Supplementary Figure S1. Assembly statistics of the 386 MRSA genomes. (a) Number of contigs. (b) N50

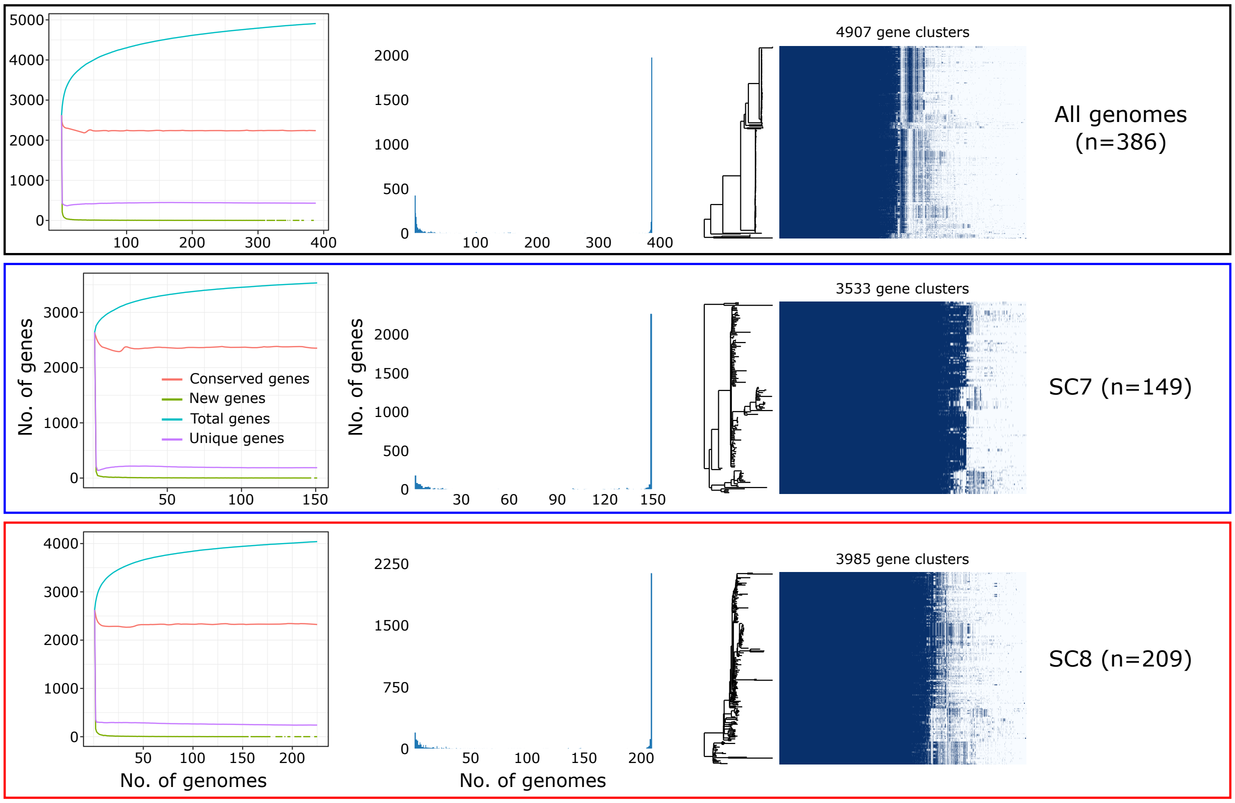

Supplementary Figure S2. Pan-genome analysis of 323 MRSA genomes. (Left) The size of the pan-genome (teal), core genome (orange), unique gene additions (purple) and new genes (green) as related to the number of individuals in the population. (Center) Gene frequency histogram indicating the number of genomes each gene is present in. (Right) Presence-absence matrix of gene clusters aligned to the phylogeny. Blue indicates presence.

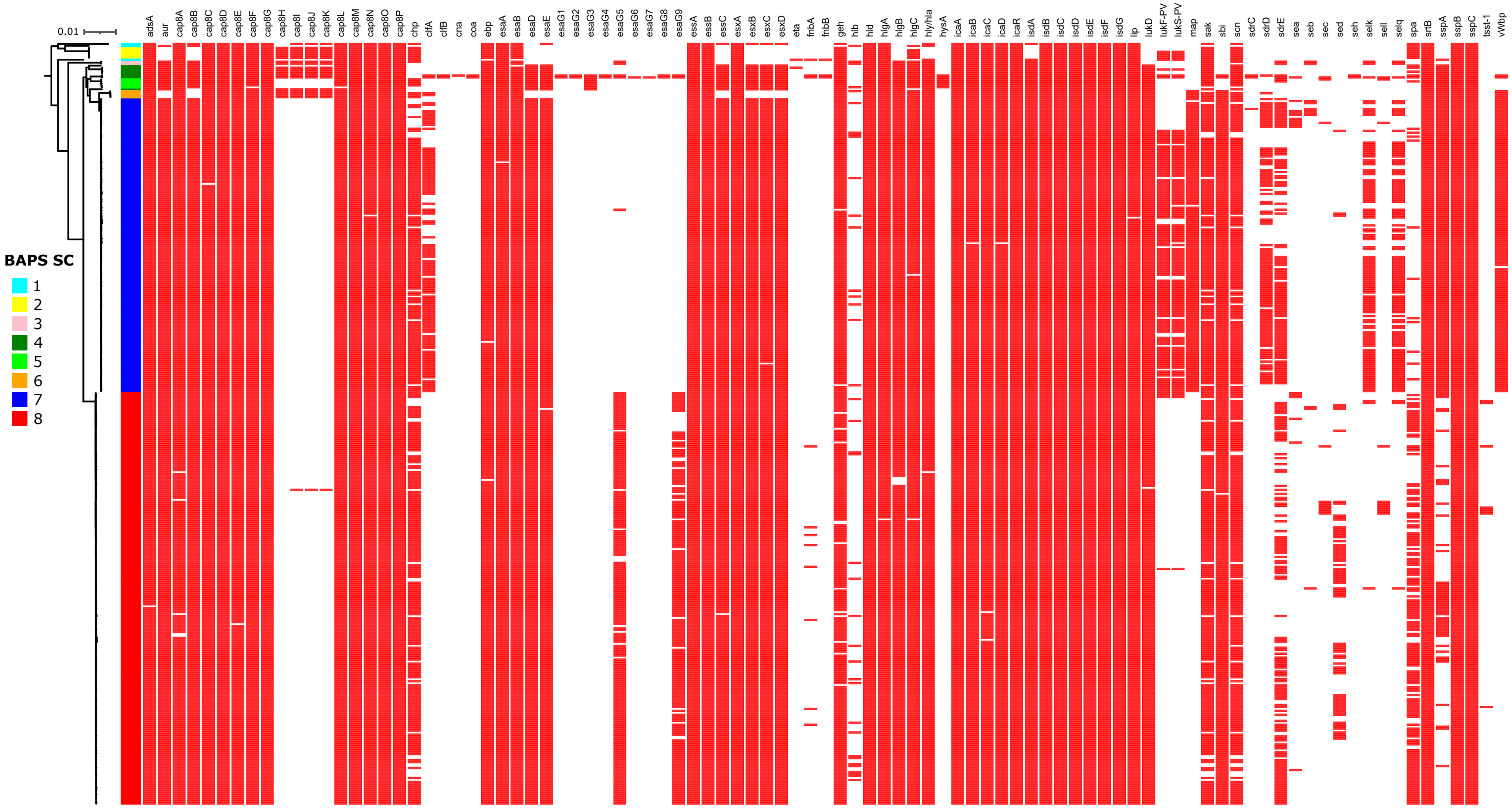

Supplementary Figure S3. Distribution of virulence genes. Details are shown in Supplementary Table S3.

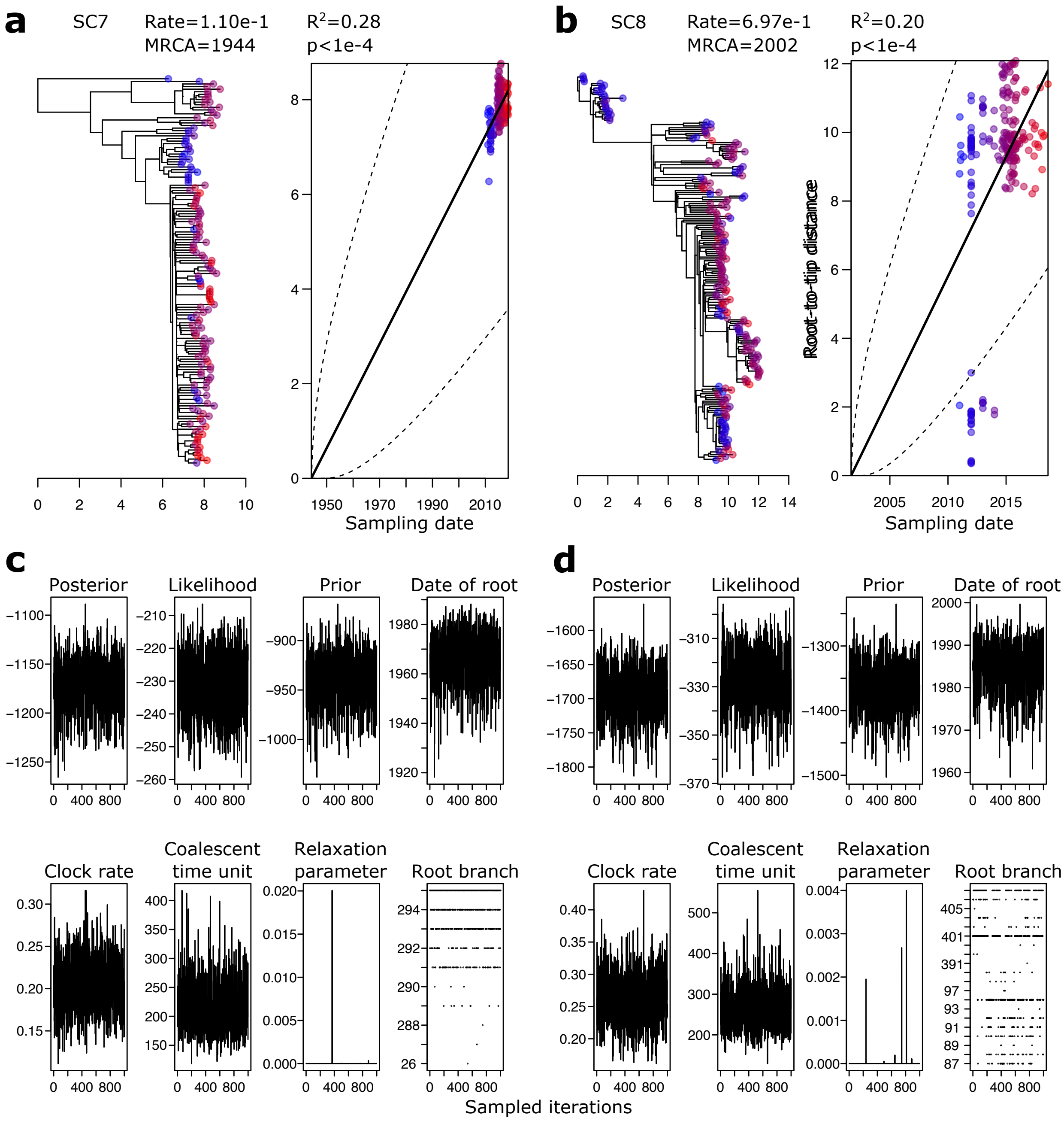

Supplementary Figure S4. Bactdating statistical tests and MCMC trace plots. (a, b) Initial rooted phylogenies and correlation test between date and root-to-tip distance within the phylogeny for sequence clusters 7 and 8. Colors of dots on tips of branches represent the date of isolation, with red representing more recent isolates and blue for older isolates. (c, d) Bactdating trace plots constructed by periodic sampling over the MCMC runs for sequence clusters 7 and 8.
